# Supplementary material for: Insect mimicry of plants dates back to the Permian
Source: Nat Commun. 2016 Dec 20;7:13735. doi: 10.1038/ncomms13735 (PMC5187432; doi:10.1038/ncomms13735)
Supplement: Supplementary Information — Supplementary Figures 1 - 10, Supplementary Tables 1 - 3, Supplementary Notes 1 - 3 and Supplementary References [file ncomms13735-s1.pdf]

Supplementary Figures

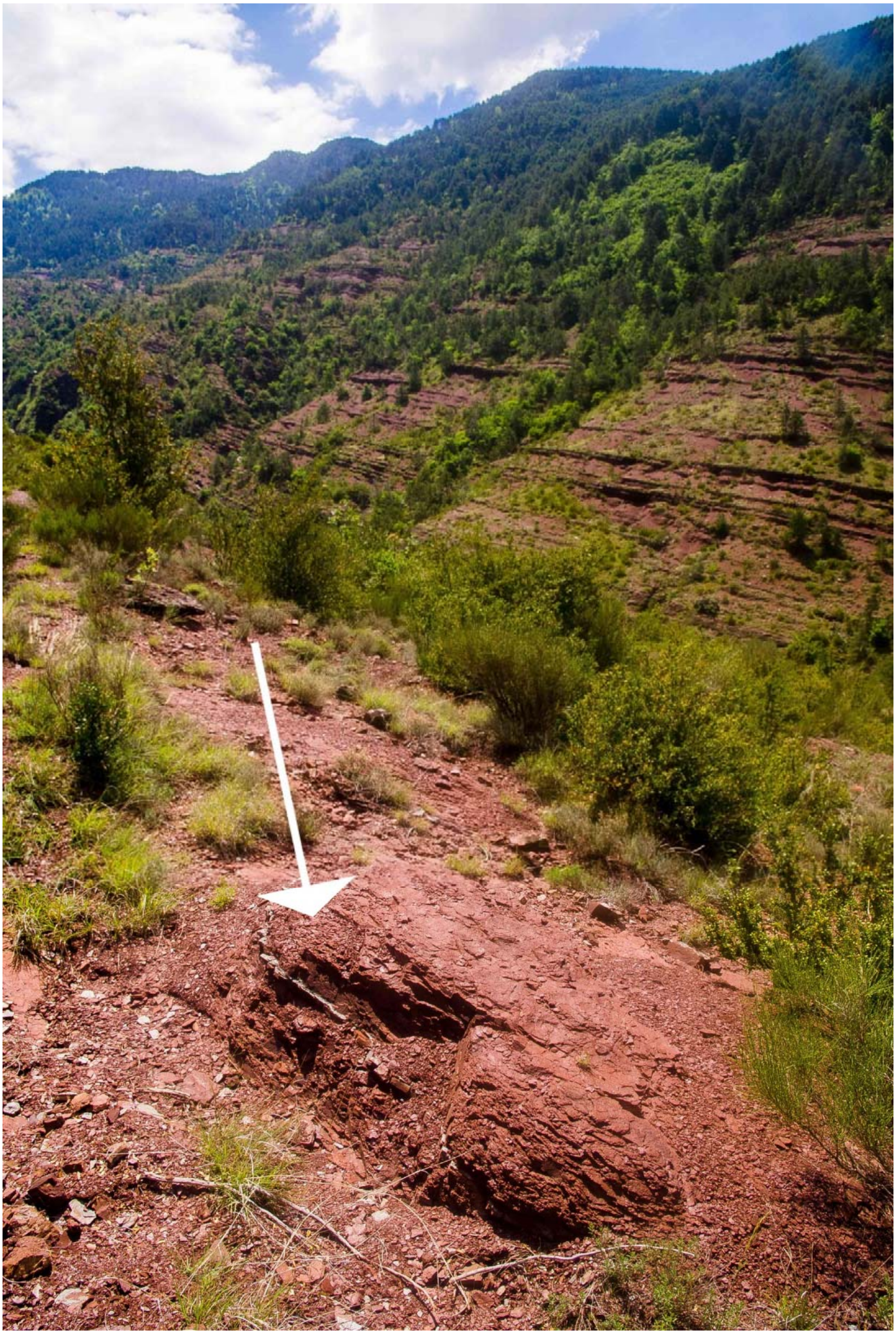

Supplementary Figure 1 | The Roua valley outcrop (alt. 760 m). Cians Formation, Dôme de Barrot. Place of discovery of *Permottetigonia gallica* (arrow). Thickness of visible deposit ca. 500 m (alt. 700 m to 1200 m).

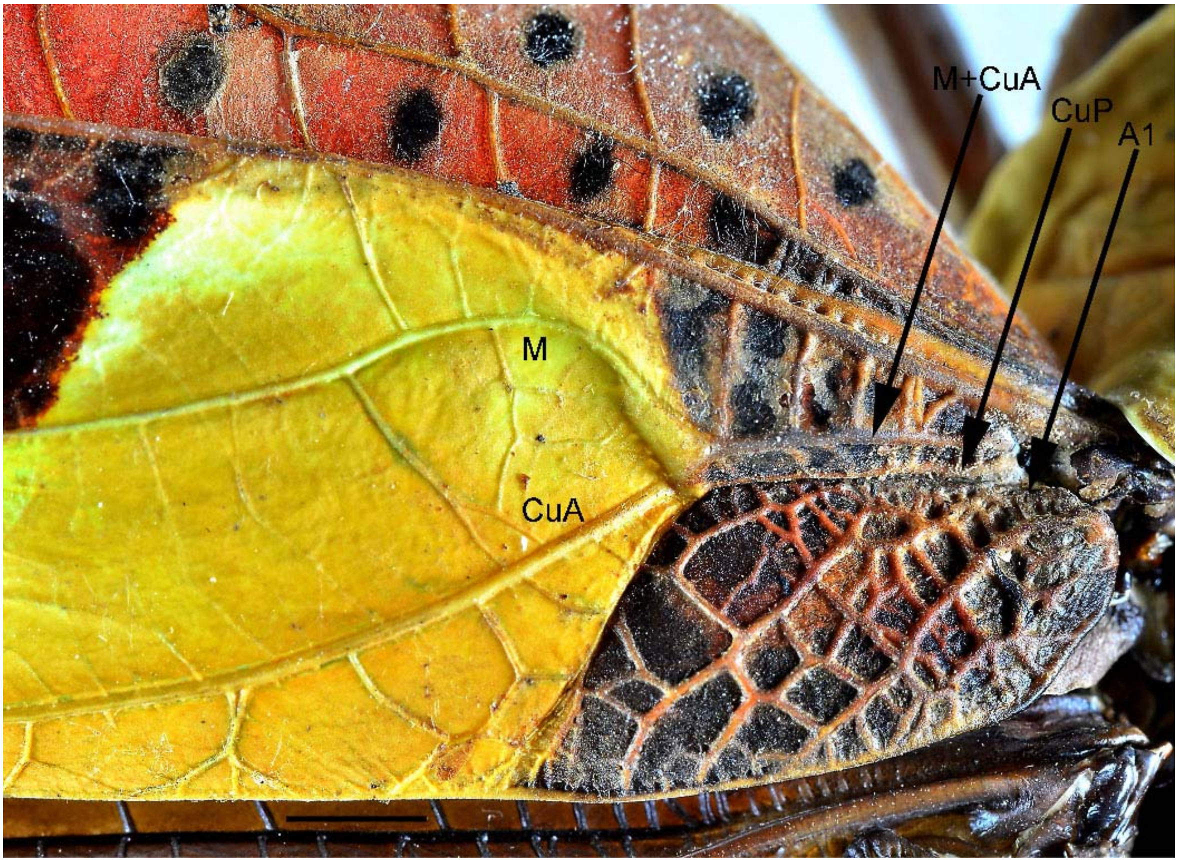

Supplementary Figure 2 | *Sanaa imperialis* (White, 1846). Tegmen base. M median vein, CuA convex cubitus anterior, CuP concave cubitus posterior, A1 convex first anal vein. Scale bar, 2 mm.

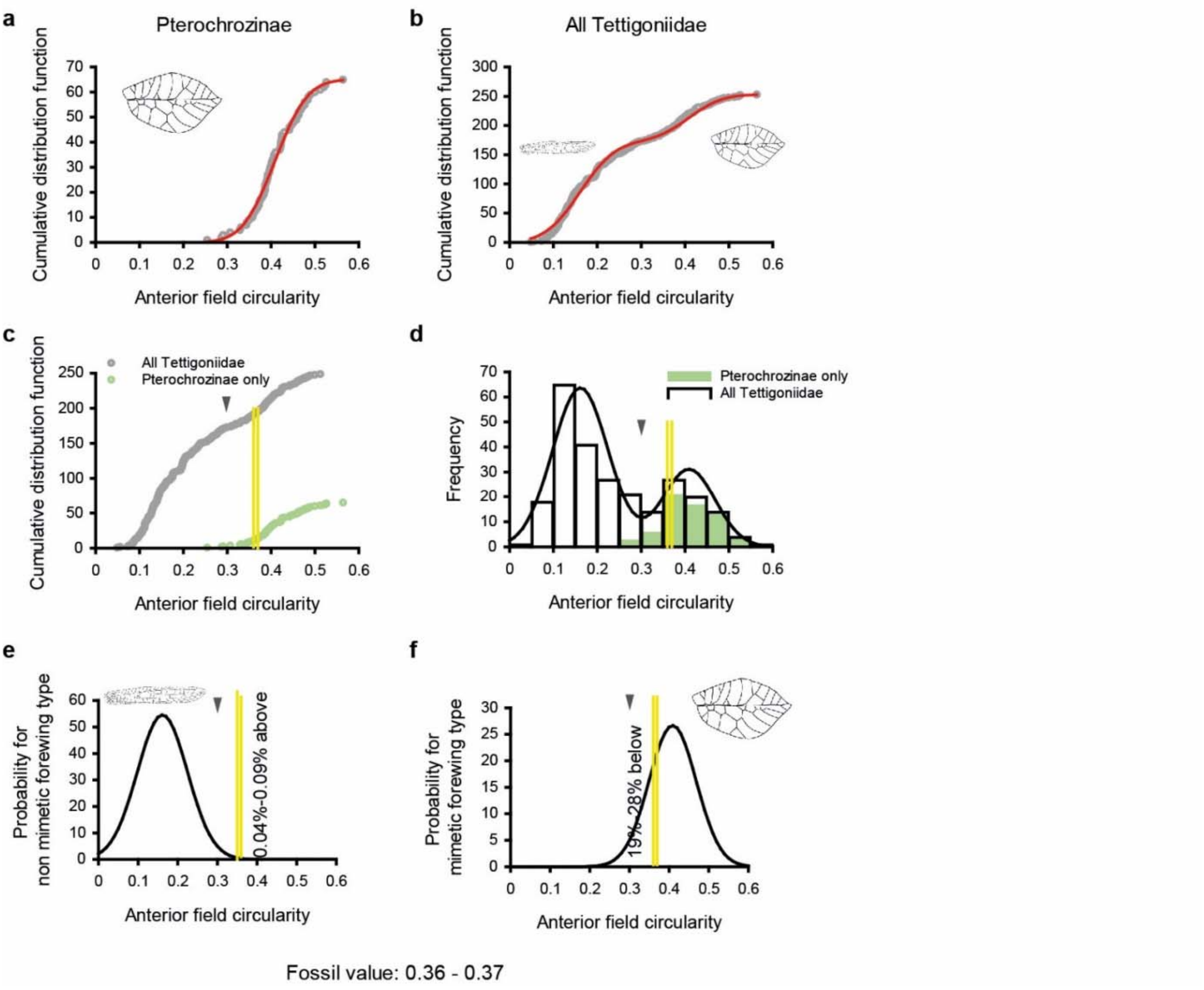

Supplementary Figure 3 | Morphometric descriptor: anterior field circularity. Pterochrozinae used to epitomize modern leaf-mimicking katydids. (**a**) Cumulative distribution of morphometric descriptors from Pterochrozinae (in grey) fitted with a single component function (in red) (equation 1). (**b**) Cumulative distribution of corresponding morphometric descriptors of all analyzed taxa fitted with a two component function (in red), with mean of one component forced to that obtained from Pterochrozinae. (**c**) Two cumulative distributions of morphometric descriptors from Pterochrozinae (in green) and all analyzed Tettigoniidae (in grey) represented at same scale together with values from *Permotettigonia gallica* (yellow bars). (**d**) Histogram constructed with cumulative distributions illustrated in (a-c); black curve corresponds to two components fitting of cumulative distribution illustrated in (b) and converted to frequency distribution. (**e**) Distribution of parameters from non-leaf-mimicking species, extracted from first component of curve in (d). (**f**) Distribution of parameters from leaf-mimicking species, extracted from second component of curve in (d). This component has a mean forced to values obtained from Pterochrozinae alone (red curve in a). Values for *P. gallica* are indicated by yellow bars, and values for *A. fossilis* are indicated by grey triangles.

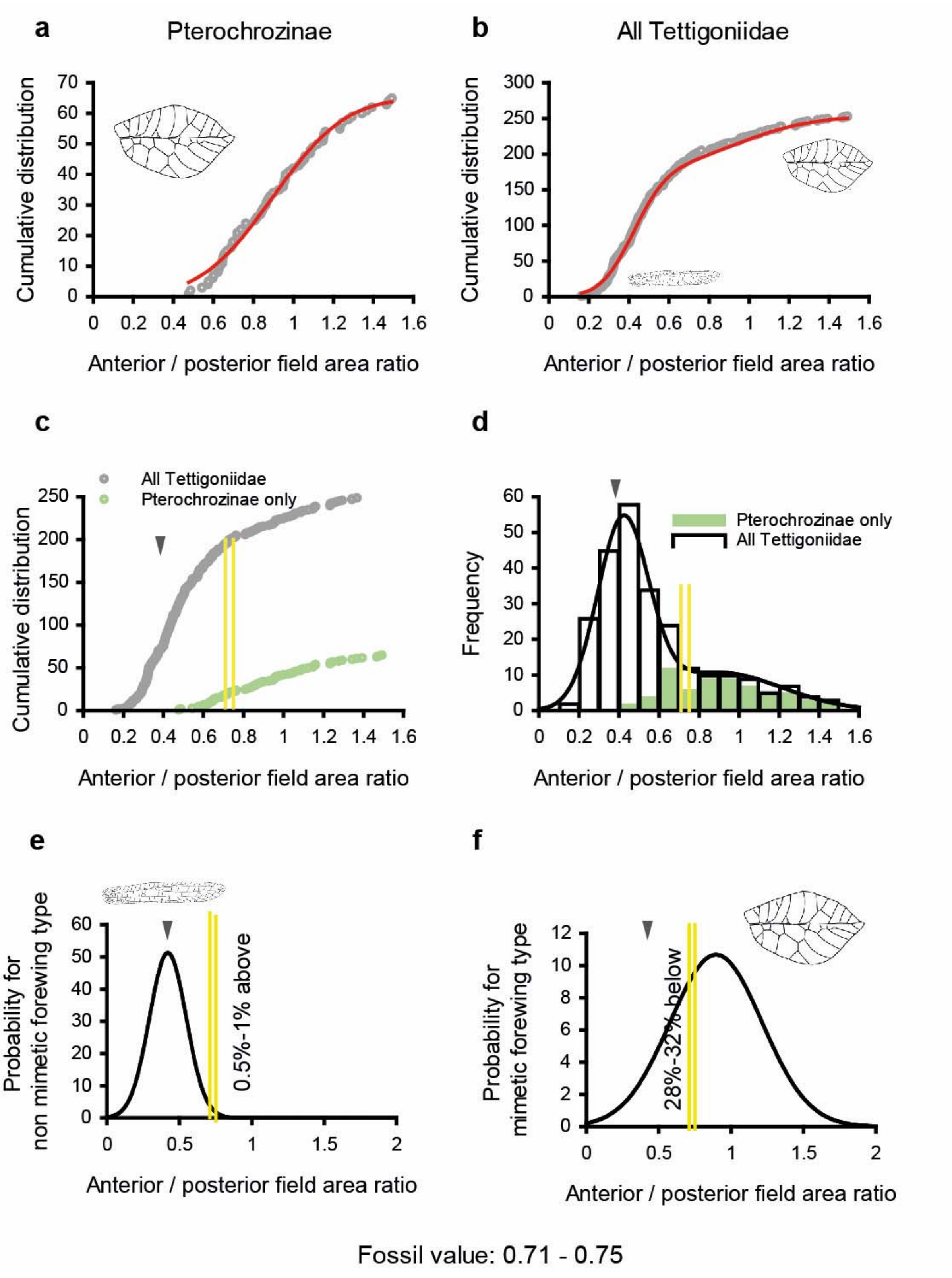

Supplementary Figure 4 | Morphometric descriptor: (anterior field area /posterior field area) ratio. Same legend as for Supplementary Figure 3.

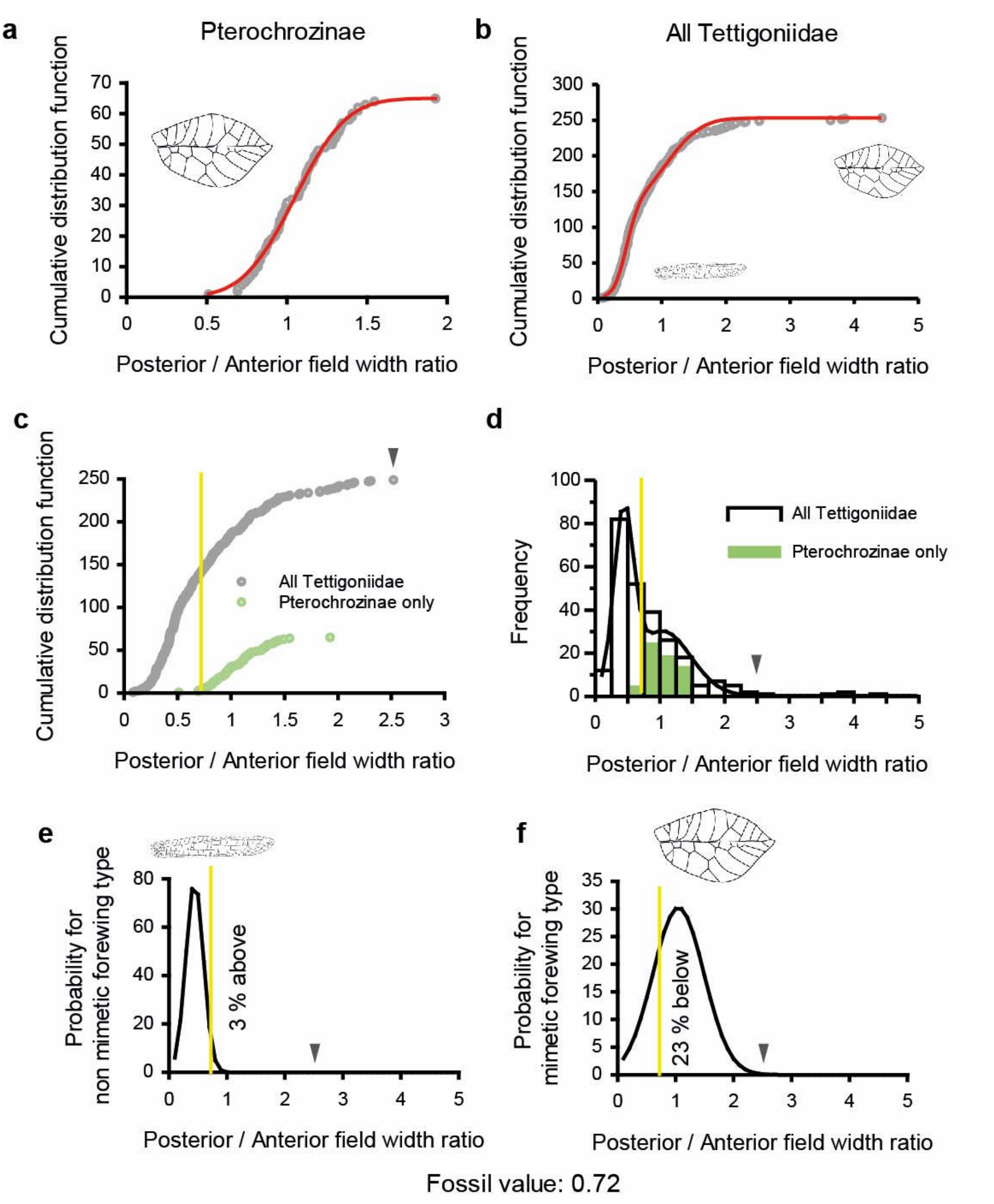

Supplementary Figure 5 | Morphometric descriptor: (posterior field width /anterior field width) Ratio. Same legend as for Supplementary Figure 3.

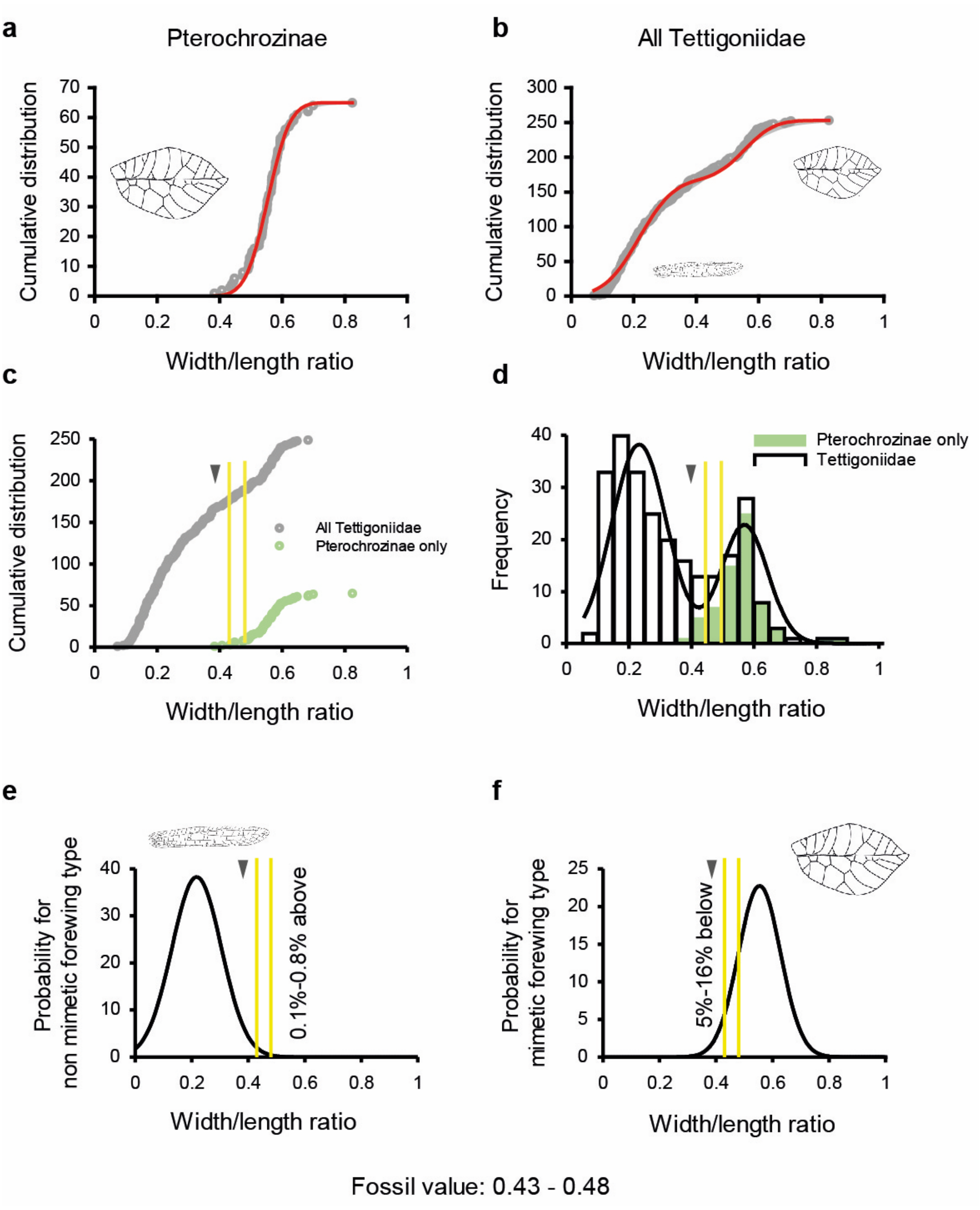

Supplementary Figure 6 | Morphometric descriptor: ratio (width/length). Same legend as for Supplementary Figure 3.

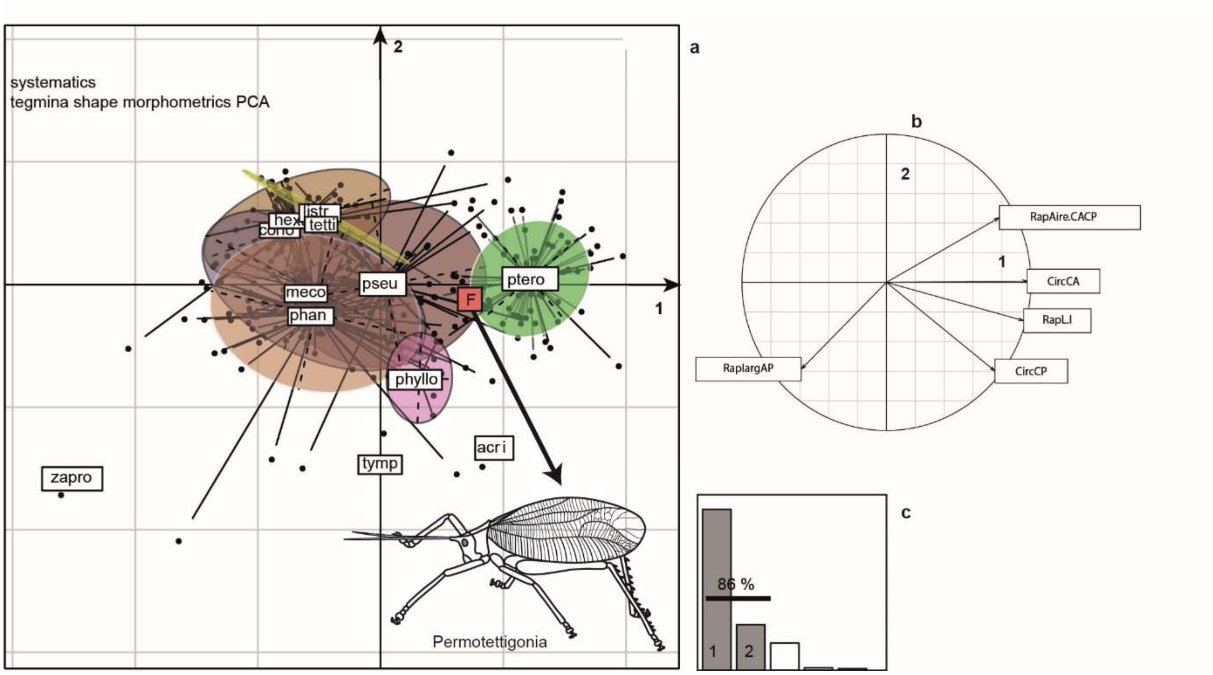

Supplementary Figure 7 | Systematics tegmina shape morphometrics (PCA). a. Factorial map on the plan 1-2 (x= axis 1, y= axis 2) organized by subfamilies. Confidence ellipses: 67% of the taxa in each subfamily. Labels of subfamilies located at their centroids. acri Acridoxeninae, cono Conocephalinae, hex Hexacentrinae, list Listroscelidinae, meco Mecopodinae, phan Phaneropterinae, phyllo Phyllophorinae, pseu Pseudophyllinae, ptero Pterophyllinae, tymp Tympanophorinae, tetti Tettigoniinae, zapro Zaprochilinae. F Fossil. **b**. Correlation circle of morphometric indices in plan 1-2 of the PCA. CircCA anterior field circularity, CircCP posterior field circularity, RapL.l (width/length) ratio, RaplargAP (anterior field width/posterior field width) ratio, RapAire.CACP (posterior field surface/anterior field surface) ratio. **c**. Eigenvalues of PCA, showing clear domination of axis 1 and axis 2 (86% of total inertia).

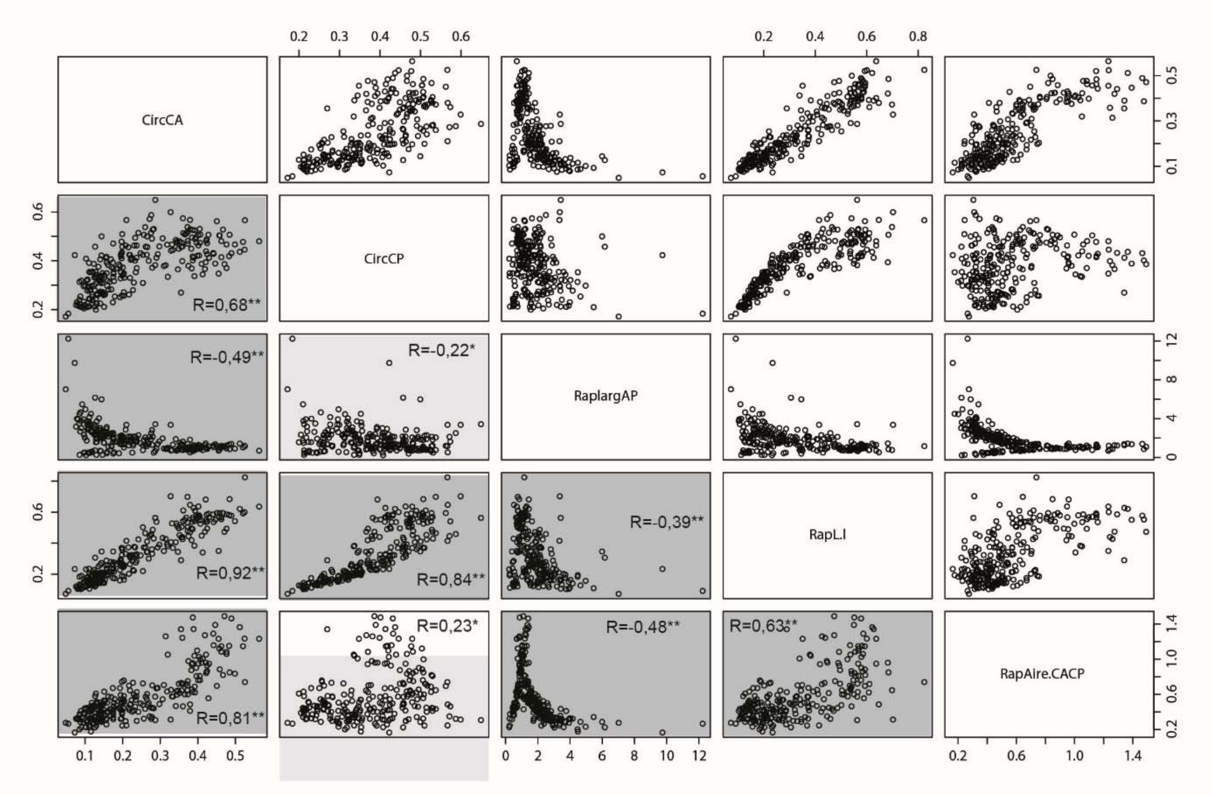

Supplementary Figure 8 | Correlations between variables. Bivariate relationships between the morphometric variables using Pearson correlation test, statistical significance indicated by the grey levels. R correlation value, * significant (0.01<p<0.001), ** highly significant (<0.001). CircCA anterior field circularity, CircCP posterior field circularity, RapL.l (width/length) ratio, RaplargAP (anterior field width/posterior field width) ratio, RapAire.CACP (posterior field surface /anterior field surface) ratio.

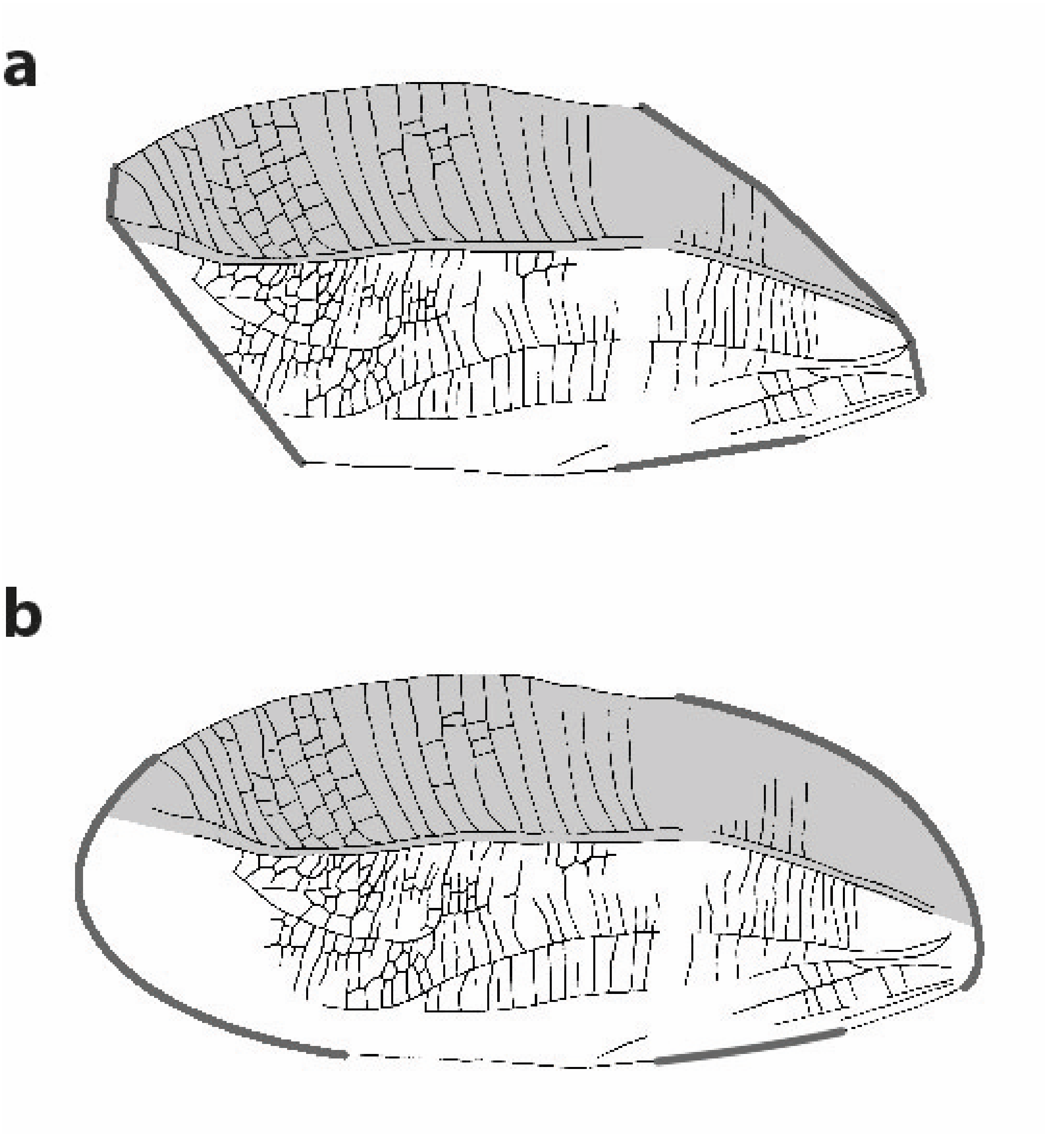

Supplementary Figure 9 | Extrapolations of missing parts of *Permotettigonia gallica*. (a) Measurements taken directly on the fossil, with no extrapolation and linear joining of missing parts. (b) Measurements taken with missing parts extrapolated with circle segment connections. Anterior field area in grey.

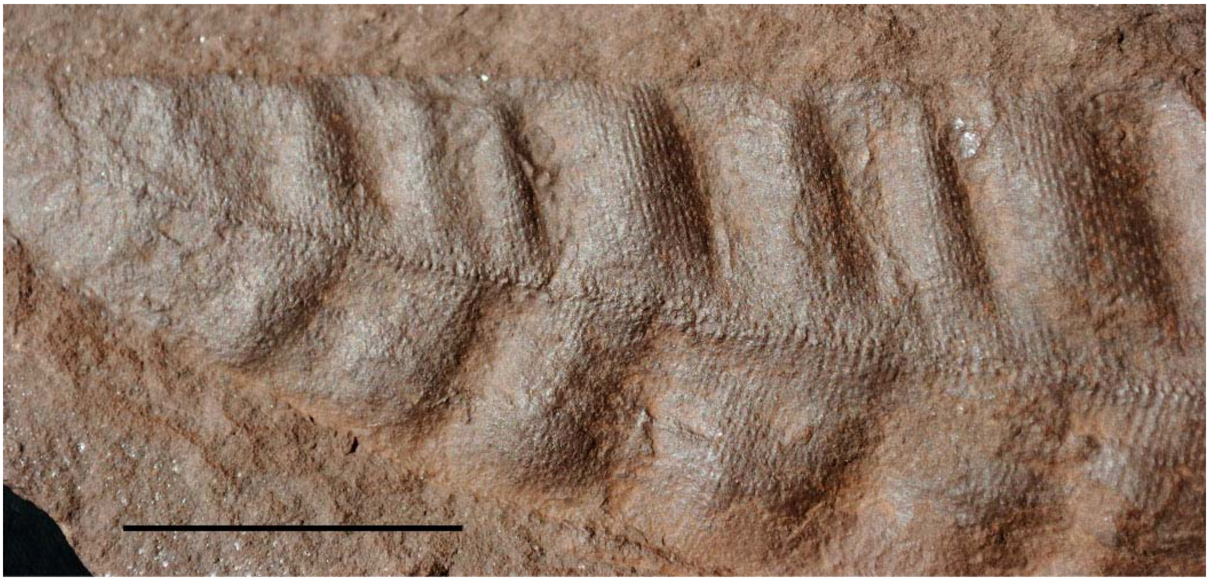

Supplementary Figure 10 | *Taeniopteris* sp. from Lodève. Leaf with intact apical region and narrow midvein, subperpendicular second order veins, and marked corrugations; specimen MULOD 1236 (Lodève Museum, France). Scale bar, 2 mm.

Sample name	Mugl.	Subfamily	GEOMETRY	PF/AF	W/L	AFA/PFA
Velella cruenta	0	Phaneropterinae	0,103	4,431	0,232	0,225
Phaneroptera nana	0	Phaneropterinae	0,130	3,804	0,192	0,246
Cratioma aberratum	1	Pseudophyllinae	0,144	0,167	0,346	0,286
Cnemidophyllum sp	1	Phaneropterinae	0,175	2,518	0,346	0,316
Phaneroptera falcata	0	Phaneropterinae	0,147	3,851	0,203	0,324
Leptotettix voluptarius	1	Pseudophyllinae	0,125	0,324	0,204	0,336
Leptotettix pubiventris	1	Pseudophyllinae	0,144	0,414	0,212	0,354
Conocephalus fuscus	0	Conocephalinae	0,111	1,896	0,143	0,364
Platychiton brunneus	1	Pseudophyllinae	0,139	0,468	0,202	0,371
Tylopsis lilifolia	0	Phaneropterinae	0,097	2,096	0,143	0,372
Xiphidiopsis lita	0	Meconematinae	0,086	3,628	0,112	0,374
Brachyphisis nattecantor	0	Meconematinae	0,158	2,308	0,237	0,395
Steirodon dentiferum	1	Phaneropterinae	0,225	2,015	0,285	0,402
Platycleis sabulosa	0	Tettigoniinae	0,112	2,084	0,126	0,407
Seselphisis visenda	0	Meconematinae	0,109	2,136	0,131	0,413
Stilpnochlora marginella	1	Phaneropterinae	0,231	0,462	0,376	0,416
Chondrosternum triste	1	Pseudophyllinae	0,175	0,539	0,235	0,418
Ruspolia nitidula	0	Conocephalinae	0,099	1,960	0,119	0,427
Tegra viridivitta	1	Pseudophyllinae	0,170	0,897	0,237	0,431
Conocephalus iris	0	Conocephalinae	0,111	1,997	0,131	0,431
Mioacris longicauda	1	Pseudophyllinae	0,202	0,525	0,246	0,439
Platycleis albopunctata	0	Tettigoniinae	0,119	1,636	0,155	0,439
Phylloptera sp.	1	Phaneropterinae	0,190	2,286	0,278	0,445
Conocephalus cinereus	0	Conocephalinae	0,140	1,430	0,142	0,447
Peucestes cristatissimus	1	Phaneropterinae	0,215	0,461	0,373	0,448
Neoconocephalus triops	0	Conocephalinae	0,113	2,153	0,143	0,456
Mustius afzelii	1	Pseudophyllinae	0,233	0,469	0,374	0,460
Platycleis tessellata	0	Tettigoniinae	0,119	1,647	0,138	0,461
Macrochiton heros	1	Pseudophyllinae	0,176	0,424	0,232	0,464
Morsimus areatus	1	Pseudophyllinae	0,221	0,464	0,304	0,488
Pseudophyllus colosseus	1	Pseudophyllinae	0,199	0,525	0,359	0,503
Decticus verrucivorus	0	Tettigoniinae	0,177	1,829	0,216	0,504
Redtenbachus viridipennis	0	Conocephalinae	0,118	1,834	0,144	0,504
Tettigonia viridissima	0	Tettigoniinae	0,159	1,721	0,190	0,505
Decticus albifrons	0	Tettigoniinae	0,142	1,532	0,174	0,505
Pseudophyllus colosseus **Male**	1	Pseudophyllinae	0,197	0,484	0,368	0,511
Acauloplacella immunis	1	Pseudophyllinae	0,224	0,602	0,324	0,533
Xerophyllopteryx fumosa	1	Pseudophyllinae	0,196	0,575	0,272	0,540
Cnemidophyllum citrifolium	1	Phaneropterinae	0,264	0,853	0,341	0,542
Pterochroza ocellata	1	Pterochrozinae	0,307	0,511	0,433	0,543
Olcinia pallidifrons	1	Pseudophyllinae	0,201	0,477	0,262	0,544
Phyllopectis crepitans	1	Pseudophyllinae	0,428	0,892	0,589	0,554
Nannonotus alatus	0	Pseudophyllinae	0,170	0,835	0,214	0,567
Mioacris javanum **Femelle**	1	Pseudophyllinae	0,233	0,599	0,324	0,569
Neoconocephalus affinis	0	Conocephalinae	0,127	1,361	0,120	0,578
Acauloplacella insularis	1	Pseudophyllinae	0,237	0,503	0,329	0,580
Tympanoptera angustipennis	1	Pseudophyllinae	0,195	0,648	0,253	0,591
Pterophylla baez	1	Pseudophyllinae	0,373	1,004	0,504	0,600
Tympanophyllum imperfectum	1	Pseudophyllinae	0,265	0,543	0,411	0,602

Lonchitophyllum reticulatum	1	Pseudophyllinae	0,351	0,661	0,453	0,613
Phyllomimus ampullatus	1	Pseudophyllinae	0,238	0,617	0,289	0,624
Tettigonia cantans	1	Tettigoniinae	0,319	1,164	0,392	0,632
Pterophylla beltrani	1	Pseudophyllinae	0,347	1,229	0,344	0,643
Mioacris javana	1	Pseudophyllinae	0,294	0,714	0,374	0,673
Tanusia brullaei	1	Pterochrozinae	0,451	0,832	0,594	0,696
Promeca pulchellerima	1	Pseudophyllinae	0,279	0,779	0,331	0,702
Sikoriella bimaculata	1	Phaneropterinae	0,310	1,095	0,349	0,705
Roxelana crassicornis	1	Pterochrozinae	0,526	0,864	0,824	0,738
Wattenwylella dispar	1	Pseudophyllinae	0,405	0,813	0,568	0,810
Mimetica imperatrix	1	Pterochrozinae	0,467	0,870	0,594	0,819
Anommatoptera ingens	1	Pterochrozinae	0,425	1,119	0,541	0,866
Parasimodera saussurei	1	Pseudophyllinae	0,384	1,179	0,419	0,943
Mimetica pehlkei	1	Pterochrozinae	0,386	0,984	0,490	0,956
Ommatoptera pictifolia	1	Pterochrozinae	0,410	0,993	0,447	0,960
Paracycloptera grandifolia	1	Pterochrozinae	0,407	0,946	0,526	0,999
Typophyllum erosum	1	Pterochrozinae	0,404	0,950	0,579	1,027
Narea elongata	1	Pseudophyllinae	0,320	1,018	0,379	1,051
Porphyromma speciosa	1	Pterochrozinae	0,520	0,971	0,593	1,147
Mimetica viridifolia	1	Pterochrozinae	0,563	1,442	0,637	1,232
Simodera acutifolia	1	Pseudophyllinae	0,314	1,167	0,427	1,260
Mastophyllum scabricolle	1	Pseudophyllinae	0,355	0,732	0,290	1,341
Cycloptera speculata	1	Pterochrozinae	0,490	0,718	0,578	1,465
Typophyllum trigonium	1	Pterochrozinae	0,387	1,142	0,552	1,476
Aganacris sp.	0	Phaneropterinae	0,120	0,367	0,215	0,325
Sylvainhugiella cesairei	0	Conocephalinae	0,129	0,641	0,127	0,608
Amblycorypha oblongifolia	1	Phaneropterinae	0,198	0,469	0,324	0,377
Anaulocomera sp.	0	Phaneropterinae	0,137	0,360	0,190	0,369
Brachyphisis viettei	0	Meconematinae	0,158	0,319	0,228	0,326
Caulopsis sp.	0	Conocephalinae	0,049	0,142	0,072	0,274
Cnemidophyllum lineatum	1	Phaneropterinae	0,169	0,402	0,279	0,354
Conocephalus fasciatus	0	Conocephalinae	0,104	0,578	0,124	0,446
Cophipora cornuta	1	Conocephalinae	0,198	0,757	0,189	0,744
Cophipora longicauda	1	Conocephalinae	0,174	0,634	0,176	0,670
Eurycorypha prasinata	1	Phaneropterinae	0,195	0,547	0,287	0,419
Holochlora biloba	1	Phaneropterinae	0,161	0,460	0,261	0,403
Meconema thalassinum	0	Meconematinae	0,203	0,683	0,260	0,508
Moncheca sp.	0	Conocephalinae	0,132	0,387	0,177	0,499
Neoconocephalus ensiger	0	Conocephalinae	0,132	0,502	0,157	0,490
Oxyprora sp.	0	Conocephalinae	0,151	0,479	0,202	0,420
Phisis holdhausi	0	Meconematinae	0,089	0,265	0,103	0,409
Pseudorhynchus lessonnii	0	Conocephalinae	0,079	0,251	0,112	0,327
Pycnopalpa sp.	1	Phaneropterinae	0,136	0,415	0,251	0,320
Ruspolia differens	0	Conocephalinae	0,099	0,489	0,116	0,442
Seselphisis sp.	0	Meconematinae	0,087	0,374	0,117	0,374
Viadana sp.	1	Phaneropterinae	0,261	0,772	0,374	0,571
Anommatoptera hoegei	1	Pterochrozinae	0,397	1,169	0,557	0,807
Anommatoptera ingens	1	Pterochrozinae	0,422	1,002	0,553	0,842
Anommatoptera maculifolia	1	Pterochrozinae	0,406	1,135	0,548	0,761
Anommatoptera ochracea	1	Pterochrozinae	0,349	1,075	0,496	0,720

Celidophylla albimacula	1	Pterochrozinae	0,329	1,408	0,407	0,649
Cycloptera arcuata	1	Pterochrozinae	0,441	0,819	0,587	1,293
Mimetica angulosa	1	Pterochrozinae	0,430	0,877	0,567	0,950
Mimetica aridifolia	1	Pterochrozinae	0,419	0,792	0,474	1,274
Mimetica casanea	1	Pterochrozinae	0,463	0,847	0,567	1,149
Mimetica imperatrix	1	Pterochrozinae	0,524	0,833	0,601	1,234
Mimetica incisa	1	Pterochrozinae	0,483	0,756	0,582	1,151
Mimetica stigmatica	1	Pterochrozinae	0,447	0,739	0,551	1,392
Mimetica subintegra	1	Pterochrozinae	0,512	0,694	0,621	1,366
Mimetica tuberata	1	Pterochrozinae	0,402	0,772	0,637	0,948
Ommatoptera elegans	1	Pterochrozinae	0,375	0,931	0,445	0,979
Ommatoptera laurifolia	1	Pterochrozinae	0,391	0,885	0,383	1,039
Ommatoptera mutila	1	Pterochrozinae	0,499	1,094	0,542	0,836
Porphyromma viridifolia	1	Pterochrozinae	0,458	1,033	0,593	0,884
Rhodopteryx hebardi	1	Pterochrozinae	0,393	1,323	0,608	0,620
Tanusia arrosa	1	Pterochrozinae	0,345	1,387	0,511	0,596
Tanusia colorata	1	Pterochrozinae	0,386	1,547	0,566	0,627
Tanusia corrupta	1	Pterochrozinae	0,343	1,443	0,528	0,632
Tanusia cristata	1	Pterochrozinae	0,331	1,301	0,527	0,575
Tanusia decorata	1	Pterochrozinae	0,357	1,281	0,504	0,645
Tanusia illusyrata	1	Pterochrozinae	0,384	1,405	0,537	0,655
Tanusia signata	1	Pterochrozinae	0,365	1,171	0,551	0,600
Tanusia undulata	1	Pterochrozinae	0,369	1,115	0,564	0,720
Tanusia versicolor	1	Pterochrozinae	0,372	1,238	0,534	0,699
Typophyllum bolivari	1	Pterochrozinae	0,287	1,490	0,563	0,573
Typophyllum champenoisi	1	Pterochrozinae	0,392	1,336	0,541	0,687
Typophyllum chlorophyllum	1	Pterochrozinae	0,379	1,927	0,647	0,485
Typophyllum cinnamum	1	Pterochrozinae	0,361	1,287	0,611	0,718
Typophyllum columbicum	1	Pterochrozinae	0,463	0,969	0,555	1,086
Typophyllum eeckei	1	Pterochrozinae	0,422	0,956	0,537	1,053
Typophyllum egregium	1	Pterochrozinae	0,401	1,125	0,578	0,762
Typophyllum erosifolium	1	Pterochrozinae	0,476	0,820	0,493	1,083
Typophyllum flavifolium	1	Pterochrozinae	0,476	1,077	0,585	1,059
Typophyllum geminum	1	Pterochrozinae	0,372	1,331	0,698	0,646
Typophyllum helleri	1	Pterochrozinae	0,398	0,925	0,567	1,098
Typophyllum histrio	1	Pterochrozinae	0,424	1,070	0,584	0,862
Typophyllum inflatum	1	Pterochrozinae	0,457	1,308	0,570	1,121
Typophyllum laciniosum	1	Pterochrozinae	0,471	0,903	0,474	1,492
Typophyllum lacinipenne	1	Pterochrozinae	0,425	1,158	0,493	0,926
Typophyllum morrisi	1	Pterochrozinae	0,421	0,692	0,682	0,960
Typophyllum mortuifolium	1	Pterochrozinae	0,485	1,185	0,684	0,850
Typophyllum mutilatum	1	Pterochrozinae	0,383	0,995	0,496	0,875
Typophyllum onkiosternum	1	Pterochrozinae	0,254	1,346	0,502	0,477
Typophyllum praeruptum	1	Pterochrozinae	0,359	1,196	0,439	1,244
Typophyllum scissifolium	1	Pterochrozinae	0,442	0,955	0,536	1,160
Typophyllum trapeziforme	1	Pterochrozinae	0,396	1,099	0,580	0,900
Typophyllum trigonum	1	Pterochrozinae	0,389	0,800	0,627	1,342
Typophyllum vignoni	1	Pterochrozinae	0,290	1,117	0,538	0,667
Abaxisotima furca	1	Phaneropterinae	0,073	0,103	0,234	0,163
Aegimia cultrifera	1	Phaneropterinae	0,280	0,473	0,487	0,445

Aegimia elongata	1	Phaneropterinae	0,242	0,434	0,424	0,400
Aegimia maculifolia	1	Phaneropterinae	0,283	1,994	0,430	0,441
Aegimia venarecta	1	Phaneropterinae	0,276	0,528	0,453	0,448
Aganacris nitida	0	Phaneropterinae	0,124	0,418	0,227	0,265
Aganacris velutina	0	Phaneropterinae	0,112	0,277	0,203	0,225
Agaurella mirabilis	1	Phaneropterinae	0,287	0,292	0,563	0,306
Anapolisia micromargaritifera	1	Phaneropterinae	0,238	0,581	0,314	0,296
Anapolisia senta	1	Phaneropterinae	0,256	0,558	0,372	0,321
Anapolisia zonata	1	Phaneropterinae	0,199	0,449	0,368	0,244
Anaulacomera libidinosa	1	Phaneropterinae	0,204	0,686	0,253	0,524
Anaulacomera pallens	1	Phaneropterinae	0,152	0,381	0,209	0,362
Ancylecha fenestrata	1	Phaneropterinae	0,260	0,487	0,466	0,434
Apoballa errabunda	1	Phaneropterinae	0,156	0,340	0,287	0,255
Apocerycta bariana	1	Phaneropterinae	0,218	0,549	0,321	0,423
Arantia hydatinoptera	1	Phaneropterinae	0,160	0,416	0,200	0,405
Arantia accrana	1	Phaneropterinae	0,199	0,578	0,248	0,391
Arantia excelsior	1	Phaneropterinae	0,184	0,604	0,264	0,473
Arantia fasciata	1	Phaneropterinae	0,248	0,688	0,321	0,496
Arantia leptocnemis	0	Phaneropterinae	0,112	0,296	0,157	0,325
Arantia orthocnemis	0	Phaneropterinae	0,140	0,502	0,209	0,387
Arantia simplicinervis	1	Phaneropterinae	0,108	0,336	0,160	0,293
Arantia fatidica	1	Phaneropterinae	0,166	0,406	0,210	0,430
Arnobia trichopus	1	Phaneropterinae	0,135	0,352	0,199	0,312
Baryprostha bellua	1	Phaneropterinae	0,272	0,470	0,446	0,347
Caedicia albidiceps	0	Phaneropterinae	0,115	0,223	0,195	0,177
Caedicia congrua	0	Phaneropterinae	0,175	0,395	0,229	0,335
Caedicia pictipes	0	Phaneropterinae	0,142	0,355	0,160	0,408
Casigneta bisinuata	0	Phaneropterinae	0,106	0,294	0,157	0,357
Casigneta cochleata	0	Phaneropterinae	0,131	0,269	0,196	0,281
Catoptropteryx capreola	0	Phaneropterinae	0,106	0,349	0,182	0,291
Catoptropteryx guttatipes	0	Phaneropterinae	0,107	0,289	0,166	0,300
Ceraia beckeri	1	Phaneropterinae	0,111	0,340	0,236	0,243
Cesasundana producta	0	Phaneropterinae	0,088	0,216	0,127	0,286
Dapanera genuteres	1	Phaneropterinae	0,175	0,481	0,276	0,373
Diogena fausta	0	Phaneropterinae	0,137	0,248	0,164	0,319
Ducetia japonica	0	Phaneropterinae	0,117	0,383	0,182	0,410
Dysmorpha obesa	1	Phaneropterinae	0,379	0,546	0,472	0,412
Dysonia diffusa	1	Phaneropterinae	0,085	0,224	0,183	0,201
Elbenia nigrosignata	0	Phaneropterinae	0,101	0,201	0,156	0,230
Elimaea (Elimaea) thaii	0	Phaneropterinae	0,076	0,315	0,121	0,323
Elimaea obtusilota	0	Phaneropterinae	0,127	0,422	0,192	0,337
Enochletica ostentatrix	1	Phaneropterinae	0,144	0,424	0,272	0,301
Euceraia atrosignata	0	Phaneropterinae	0,090	0,331	0,189	0,222
Eulioptera monticola	0	Phaneropterinae	0,114	0,362	0,168	0,295
Euthyrrhachis gracilis	0	Phaneropterinae	0,084	0,286	0,133	0,316
Holochlora paradoxa	1	Phaneropterinae	0,128	0,303	0,210	0,300
Holochlora venosa	1	Phaneropterinae	0,141	0,397	0,206	0,363
Phylloptera difficilis	1	Phaneropterinae	0,208	0,350	0,331	0,292
Hyperphrona nitidipennis	0	Phaneropterinae	0,127	0,162	0,306	0,224
Amacroxiphus nigrifrons	0	Conocephalinae	0,120	0,379	0,130	0,470

Axylus bimaculatus	0	Conocephalinae	0,151	0,669	0,143	0,630
Axylus castaneus	0	Conocephalinae	0,144	0,423	0,175	0,405
Axylus thoracicus	0	Conocephalinae	0,147	0,430	0,161	0,476
Clasma parcispinosa	0	Conocephalinae	0,131	0,437	0,151	0,426
Coniungoptera nothofagi	0	Conocephalinae	0,235	0,627	0,275	0,537
Eppia truncatipennis	0	Conocephalinae	0,153	0,428	0,166	0,520
Eppioides malaya	0	Conocephalinae	0,126	0,422	0,167	0,401
Eulobaspis moluccana	0	Conocephalinae	0,179	0,631	0,173	0,544
Eulobaspis personata	0	Conocephalinae	0,193	0,716	0,159	0,671
Mesagraecia bicolor	0	Conocephalinae	0,230	0,707	0,222	0,597
Paroxylakis setosa	0	Conocephalinae	0,254	0,804	0,220	0,724
Pyrgocorypha hamata	0	Conocephalinae	0,129	0,515	0,136	0,464
Salomona affine	0	Conocephalinae	0,217	0,802	0,202	0,699
Scytocera longicornis	0	Conocephalinae	0,203	0,971	0,185	0,756
Subria grandis	0	Conocephalinae	0,131	0,631	0,141	0,515
Acridoxena hewaniana	1	Acridoxeninae	0,328	0,297	0,702	0,311
Afromecopoda preussiana	0	Mecopodinae	0,104	0,272	0,177	0,291
Albertisiella acanthodiformis	1	Mecopodinae	0,193	0,458	0,227	0,435
Anoedopoda erosa	1	Mecopodinae	0,156	0,392	0,257	0,309
Anoedopoda lamellata	1	Mecopodinae	0,141	0,331	0,261	0,313
Biroa atrospinosa	0	Mecopodinae	0,096	0,254	0,150	0,275
Characta bituberculata	1	Mecopodinae	0,195	0,574	0,297	0,320
Euhexacentrus annulicornis	1	Hexacentrinae	0,228	0,818	0,227	0,732
Ityocephala francoisi	1	Mecopodinae	0,197	0,464	0,273	0,328
Mecopoda platyphoea	1	Mecopodinae	0,266	0,635	0,344	0,482
Pachysmopoda abbreviata	0	Mecopodinae	0,334	0,761	0,369	0,572
Paralistroscelis listrosceloides	0	Listroscelidinae	0,204	0,745	0,165	0,580
Parateuthras truncatus	1	Phaneropterinae	0,128	0,434	0,158	0,346
Segestes beieri	0	Hexacentrinae	0,097	0,426	0,122	0,390
Tympanophora splendida	1	Tympanophorinae	0,210	0,298	0,415	0,246
Zaprochilus mongabarra	0	Zaprochilinae	0,055	0,082	0,091	0,266
Agraecia punctata	0	Conocephalinae	0,150	0,652	0,144	0,530
Diophanes rosescens	0	Pseudophyllinae	0,219	0,640	0,264	0,644
Iaratrox maculata	0	Conocephalinae	0,147	0,509	0,145	0,491
Kevaniella bipunctata	0	Phaneropterinae	0,146	0,420	0,165	0,481
Lea floridensis divergens	1	Pseudophyllinae	0,298	0,605	0,390	0,489
Starkonsa nigrifrons	0	Conocephalinae	0,141	0,492	0,149	0,469
Steirodon dentatum	1	Phaneropterinae	0,174	0,506	0,265	0,401
Temnophyllus atrosignatus	1	Pseudophyllinae	0,219	0,489	0,312	0,480
Temnophyllus speciosus	1	Pseudophyllinae	0,197	0,441	0,269	0,418
Trigonocorypha angustata	1	Phaneropterinae	0,162	0,884	0,214	0,575
Trigonocorypha buettikeri	1	Phaneropterinae	0,145	0,479	0,193	0,474
Trigonocorypha tiahmae	1	Phaneropterinae	0,269	0,725	0,300	0,501
Corycus sp.	1	Mecopodinae	0,361	0,612	0,541	0,462
Lesina ensifera	0	Conocephalinae	0,455	1,443	0,350	1,233
Cesacomora cucullata	1	Conocephalinae	0,413	1,335	0,339	1,007
Phaneroptera magna	0	Phaneropterinae	0,124	0,297	0,167	0,266
Phaneroptera minima	0	Phaneropterinae	0,112	0,240	0,140	0,322
Tylopsis dispar	0	Phaneropterinae	0,093	0,183	0,109	0,340
Tylopsis irregularis	0	Phaneropterinae	0,081	0,255	0,105	0,315

Alloteratura longicauda	0	Meconematinae	0,112	0,292	0,107	0,434
Phyllophorella inermis	1	Phyllophorinae	0,278	0,356	0,361	0,341
Phyllophorella woodfordi	1	Phyllophorinae	0,342	0,528	0,406	0,466
Sasima amplifolia	1	Phyllophorinae	0,276	0,399	0,459	0,321
***Permotettigonia gallica* A**	?	?	0,373	1,395	0,477	0,750
***Permotettigonia gallica* B**	?	?	0,359	1,337	0,426	0,715
Archepseudophylla fossilis	?	?	0,300	2,528	0,392	0,391

Supplementary table 1. Dataset for the morphometric analyses. *Permotettigonia gallica* A: linear extrapolation; *Permotettigonia gallica* B: circular extrapolation; Mugleston et al criterion: 0: thorax height larger than FW width; 1: FW wider than thorax height; Geometry: Anterior field circularity; ratio pf/af: posterior field width/anterior field width; Ratio W/L: width/length; Ratio afa/pfa: anterior field area/posterior field area

	Models with one component			Models with two components				Comparison of two component models with one component models		
	Model	SS	df	Non-leaf-mimicking component	Leaf-mimicking component	SS	df	P	Reduction in AICc	Reduction in BIC
Width/length ratio	Mean (A3): 0.32<br>SD (A4): 0.20	50512	251	Weight (A5): 0.66<br>Mean (A3): 0.22<br>SD (A4): 0.09	Weight (1-A5): 0.34<br>Mean (A1): 0.55<br>SD (A2): 0.07	7074	249	>0.9999	489	475
Posterior / Anterior field width ratio	Mean (A3): 0.44<br>SD (A4): 0.15	44754	251	Weight (A5): 0.47<br>Mean (A3): 0.44<br>SD (A4): 0.15	Weight (1-A5): 0.53<br>Mean (A1): 1.05<br>SD (A2): 0.44	3687	249	>0.9999	623	609
Anterior / posterior field area ratio	Mean (A3): 0.42<br>SD (A4): 0.13	47054	251	Weight (A5): 0.66<br>Mean (A3): 0.42<br>SD (A4): 0.13	Weight (1-A5): 0.34<br>Mean (A1): 0.90<br>SD (A2): 0.32	2434	249	>0.9999	741	727
Anterior field circularity	Mean (A3): 0.16<br>SD (A4): 0.06	54819	251	Weight (A5): 0.68<br>Mean (A3): 0.16<br>SD (A4): 0.06	Weight (1-A5): 0.32<br>Mean (A1): 0.41<br>SD (A2): 0.06	8327	249	>0.9999	469	455

Supplementary table 2. Comparison of non-linear regression models. Model with one component (equation 1) compared to model with two components (equation 2) with the mean (A1) fixed to the value obtained for the Pterochrozinae, epitomizing leaf-mimicking katydids. SS: sum of squares; df: degree of freedom; P: probability that the data are better described with the two component model; dAICc: difference in corrected Akaike information criterion between the two models (decrease in AICc with two components model); dBIC: difference in Bayesian information criterion between the two models (decrease in BIC with two components model).

	**Modeling** with modern katydids		**Measurements** fossil		**Comparison** fossil / modeling	
	Non leaf mimicking component	Leaf mimicking component	Linear extrapolation	Circular extrapolation	% of non-leaf-mimicking distribution ≥ fossil value	% of leaf-mimicking distribution ≤ fossil value
Width/length ratio	Weight (A5): 0.66<br>Mean (A3): 0.22<br>SD (A4): 0.09	Weight (1-A5): 0.34<br>Mean (A1): 0.55<br>SD (A2): 0.07	0.48	0.43	0.1 % - 0.8 %	5 % - 16 %
Posterior / Anterior field width ratio	Weight (A5): 0.47<br>Mean (A3): 0.44<br>SD (A4): 0.15	Weight (1-A5): 0.53<br>Mean (A1): 1.05<br>SD (A2): 0.44	0.72	0.72	3 %	23 %
Anterior / posterior field area ratio	Weight (A5): 0.66<br>Mean (A3): 0.42<br>SD (A4): 0.13	Weight (1-A5): 0.34<br>Mean (A1): 0.90<br>SD (A2): 0.32	0.75	0.71	0.5 % - 1 %	28 % - 32 %
Anterior field circularity	Weight (A5): 0.68<br>Mean (A3): 0.16<br>SD (A4): 0.06	Weight (1-A5): 0.32<br>Mean (A1): 0.41<br>SD (A2): 0.06	0.37	0.36	0.04 % - 0.09 %	19 % - 28 %

Supplementary table 3. Comparison of measurements taken on *Permotettigonia gallica* and modeling of modern katydid wings. The parameters obtained with nonlinear regression (*A1-A5*) correspond to the parameters given in *Equation 2*.

SUPPLEMENTARY NOTES

Supplementary Note 1 | Locality and horizon

The fossil was discovered by one of us (P.R.) in the Dôme du Barrot, 760 m alt., in the Cians Formation (Red Permian), Roua valley, near the village of Daluis, close to Var River, Alpes Maritimes, France (Supplementary Fig. 1). It is the third fossil insect found in this Formation. The first one is a griffenfly (Odonatoptera: Meganeuridae) (Collet du Brec, Léouvé)[1-2]. The second one is an undetermined insect also found at the Collet du Brec[3]. One of us (R.G.) has discovered in 2015 some Notostraca, Ostracoda, and arthropod ichnites in the Collet du Brec area, otherwise very poor in fossils. The Cians Formation is of continental origin constituted of red mudstones and sandstones issued from the erosion of the Variscan relief[4]. A Wordian or Roadian age (269-265 Ma) was proposed for the Léouvé Formation, which is above the Cians Formation[3,5]. More recently, a Roadian age (272.5-268 Ma) was proposed for the red Permian of the Dome de Barrot[6].

Supplementary Note 2 | Systematic Paleontology: Extended descriptions and comments

Since all taxa are monotypic, the included species and genus are automatically herein designated as the type species and type genus for the associated generic and familial names.

Insecta Linné, 1758

Orthoptera Olivier, 1789

Ensifera Chopard, 1920

Tettigonioidea Krauss, 1902

Family Permotettigoniidae Nel et Garrouste, fam. nov.

Genus *Permotettigonia* Nel et Garrouste, gen. nov.

Permotettigonia gallica Nel et Garrouste, sp. nov.

2007 'archaeorthoptère de l'ordre des Titanoptera' (O. Béthoux, pers. comm.)[3]

Holotype. MNHN-LP-R 63853, Muséum National d'Histoire Naturelle, Paris, France.

Etymology. The genus name refers to Permian and *Tettigonia*; the species name refers to the Latin name for France.

Diagnosis. The fossil is a nearly complete and very broad tegmen (fore wing) with a very broad corrugate field between subcostal vein ScP and anterior wing margin, crossed by numerous long veinlets alternatively convex and concave, all nearly perpendicular to ScP and to anterior wing margin. A second broad field is present between radial vein R and median vein M, and a third between M and cubital complex vein CuA+CuPaα; all crossed by long veinlets more or less curved and perpendicular to main longitudinal veins; M and CuA+CuPaα simple and distally fused again; CuP divided into branches CuPaα, CuPaβ, and CuPb.

Description. This fossil is the imprint in rock of the antero-basal part (ca. four-fifth of wing) of a fore wing; no trace of coloration preserved; it is impossible to determine whether it is a right or left wing. Length of fragment 33.0 mm, width 15.0 mm; probable length of wing ca. 40 mm; presence of an enigmatic zone near bases of radius and ScP that could correspond to costal field between a possible vein ScA and costal margin, rather short and broad, 7.0 mm long, 3.0 mm wide, with phantom-like traces of veins; concave vein ScP closely parallel to convex R (distally RA), diverging near their apices; field between ScP and anterior wing margin very broad and long, 26.0 mm long, 5.5 mm wide, crossed by a series of about 26 alternatively concave and convex veins, all straight, and nearly perpendicular to ScP and anterior wing margin, distance between two such concave veins ca. 2 mm; presence of

parallel longitudinal short crossveins present between these veinlets, defining small quadrangular cells; RP emerging from R far from wing base, simple in its preserved part, making a deep posterior curve and directed distally towards RA; presence of a secondary longitudinal vein between RP and RA, not directly branching on RA; M+CuA separating from radius near wing base; a broad and elongate field between R and M, 25.0 mm long, 4.0 mm wide, crossed by a series of long veinlets more or less perpendicular to R and M and a rather irregular archedictyon between these veinlets; concave M and convex CuA separated ca. 2.0 mm from wing base; an elongate and narrow field between M and CuA, 18.0 mm long, 3.0 mm wide; M and CuA fused again 24.0 mm from wing base; concave CuP divided into two branches CuPa and CuPb near wing base; CuPa separating into CuPaα and CuPaβ ca. 5 mm distal of wing base; CuPaα short and ending on CuA; CuPaβ and CuPb simple and nearly straight; a simple and straight first anal vein posterior to CuPb, other anal veins not preserved or absent; field of CuP – anal vein very narrow and reduced compared to the rest of the wing, ca. 14 long and 2 mm wide, nearly perpendicular to the main plan of the wing surface.

Discussion. The pattern of venation of *Permotettigonia* corresponds to that of an Orthoptera in the basally fused veins R, M and CuA, presence of M+CuA, CuP with two main branches CuPb and CuPa, the latter being subdivided into a short CuPaα distally ending into CuA, and a long CuPaβ[7]. *Permotettigonia* displays all the synapomorphic characters of the Tettigoniidae, i.e., complete absence of the vein MA, M and CuA with no secondary branches; archedictyon always present, but more or less developed[8]. *Permotettigonia* differs from the modern Tettigoniidae in the presence of a CuP subdivided into branches CuPaα, CuPaβ, and CuPb, while the latter have a CuPa distally forked or not, and no CuPb (apomorphy of the modern katydids) (see Fig. 1, Supplementary Fig. 2), the situation in *Permotettigonia* being then plesiomorphic[7]. *Permotettigonia* is not a representative of the crown group Tettigoniidae, but certainly belongs to the stem group of the Tettigoniidae. The

type tegmen of *Permotettigonia* shows no trace of stridulatory organ, suggesting it could be that of a female, or a mute male.

Supplementary Note 3 | Remarks

1) Few Paleozoic and Mesozoic Orthoptera have a broad subcostal field with a series of veinlets nearly perpendicular to ScP and costal margin:

- The Mesozoic *Deinovitimia insolita* Gorochov, 1989 (in the enigmatic family Vitimiidae Sharov, 1968). This taxon differs from *Permotettigonia* in the pectinate MP+CuA and the forked RP(+MA)[8-9];

- The Middle Permian *Raphogla rubra* Bethoux et al., 2002 (in the enigmatic family Raphoglidae Bethoux et al., 2002). This taxon differs from *Permotettigonia* in the MP+CuA, MA, and RP with several branches[10].

2) The Lower Cretaceous *Haglotettigonia* Gorochov, 1988 is, after Gorochov[11], the oldest representative of the Tettigonioidea, and unique representative of the extinct Haglotettigoniidae. Gorochov[12] justified the affinities of this taxon with the Tettigoniidae on the basis of the following characters (translated from the Russian by our colleague Alexander Kirejtshuk): 'fore wing of male of primitive structure, mostly similar to Hagloidea; stems Sc and R placed apart each other; there are remains of distal part of second branch of MA and also completely developed, but broken CuA2 with distal and proximal parts shifted relatively each other along diagonal vein; diagonal vein developed, angularly curved and with pectinate fan; between the base of distal part of CuA2 and diagonal vein there is somewhat widened cell (precursor of mirror of Tettigoniidae); strings well expressed; MP+CuA1 clearly pectinate; transverse venation normal, i.e. not transformed into a net of secondary archedictyon. These characters distinguish the new family from Tettigoniidae'.

Among these characters, only the cell 'precursor' of the mirror of Tettigoniidae would support an affinity with this family because all other characters are plesiomorphies. The problem is that *Haglotettigonia* has no real wide cell that could be considered as a mirror but a net of rather small cells in the field where the alleged mirror should be[12]. Consequently the affinities of *Haglotettigonia* with the Tettigoniidae should be verified.

3) The modern caeliferan Trigonopterygidae have also leaf-like tegmina, with several specializations convergent with the leaf-like katydids, viz. the presence of a double longitudinal vein that mimics the median vein of an angiosperm leaf, presence of a very broad subcostal field, etc. Nevertheless these Caelifera strongly differ from our fossil in the median vein that becomes fused again with radius in basal third of tegmina, and RP that is joined to RA for a long distance and emits several posterior branches before separating from RA.

SUPPLEMENTARY REFERENCES

1. Laurentiaux-Vieira, F. & Laurentiaux, D. Découverte d'un insecte protodonate dans le Permien des Alpes Maritimes. *Comptes-Rendus de l'Académie des Sciences* **257**, 3018-3020 (1963).
2. Nel, A. et al. Revision of Permo-Carboniferous griffenflies (Insecta: Odonatoptera: Meganisoptera) based upon new species and redescription of selected poorly known taxa from Eurasia. *Palaeontographica* (A) **289**, 89-121 (2009).
3. Durand, M. & Gand, G. Le Permien et le Trias du Dôme de Barrot (Alpes-Maritimes). Livret-guide de l'excursion annuelle de l'Association des Géologues du Permien et du Trias, 18-20 septembre 2007: 1-26 (2007).

4. Bourquin, S., Durand, M., Diez, J.B., Broutin, J. and Fluteau, F. 2007. The Permian-Triassic boundary and lower Triassic sedimentation in western European basins: an overview. *Journal of Iberian Geology*, **33** (2): 221-236.
5. Kruiver, P. P. et al. The implications of non-suppressed geomagnetic secular variation during the Permo-Carboniferous Reversed Superchron. *Physics of the Earth and Planetary Interiors* **131**, 225-235 (2002).
6. Haldan, M.M. et al. A comparison of detailed equatorial red bed records of secular variation during the Permo-Carboniferous Reversed Superchron. *Geophysical Journal International* **177**, 834-848 (2009).
7. Béthoux, O. & Nel, A. Venation pattern and revision of Orthoptera *sensu nov*. and sister groups. Phylogeny of Palaeozoic and Mesozoic Orthoptera *sensu nov*. *Zootaxa* **96**, 1–88 (2002).
8. Gorochov, A. V. [New taxa of the orthopteran families Bintoniellidae, Xenopteridae, Permelcanidae, Elcanidae and Vitimiidae (Orthoptera, Ensifera).] *Vestnik Zoologii* **27**, 20-27. [in Russian.] (1989).
9. Gorochov, A. V. Sistema i evolyutsiya pryamokrylykh podotryada Ensifera (Orthoptera) [System and Evolution of the suborder Ensifera (Orthoptera).] Parts 1 and 2. *Trudy Zoologicheskogo Instituta* **260**, 3-224 & **261**, 3-212. [in Russian] (1995).
10. Béthoux, O. et al. *Raphogla rubra* gen. n., sp. n., the oldest representative of the clade of modern Ensifera (Orthoptera: Tettigoniidea, Gryllidea) (Lodève Permian basin, France). *European Journal of Entomology* **99**, 111-116 (2002).
11. Gorochov, A. V. Review of Triassic Orthoptera with descriptions of new and little known taxa: part 2. *Paleontological Journal* **39**, 272-279 (2005).
12. Gorochov, A. V. [Classification and phylogeny of katidids (Gryllida = Orthoptera, Tettigonioidea).] pp. 145-190. In: Ponomarenko, A. G. (ed.). *Melovoj biotsenoticheskij*

krizis i evolutsiya nasekomykh. [Cretaceous biocoenotic crisis and insect evolution.], Nauka, Moscow, 1-230 [in Russian.] (1988).
